# Population pharmacokinetics of artemether–lumefantrine plus amodiaquine in patients with uncomplicated *Plasmodium falciparum* malaria

**DOI:** 10.1002/bcp.70301

**Published:** 2025-10-06

**Authors:** Junjie Ding, Richard M. Hoglund, Rob W. van der Pluijm, James J. Callery, Thomas J. Peto, Rupam Tripura, Sukanta Das, Nguyễn Hoàng Châu, Cholrawee Promnarate, Mavuto Mukaka, Lek Dysoley, Caterina Fanello, Marie A. Onyamboko, Anupkumar R. Anvikar, Mayfong Mayxay, Frank Smithuis, Lorenz von Seidlein, Mehul Dhorda, Chanaki Amaratunga, M. Abul Faiz, Ho Dang Trung Nghia, Nicholas J. White, Nicholas P. J. Day, Arjen M. Dondorp, Joel Tarning

**Affiliations:** ^1^ Mahidol Oxford Tropical Medicine Research Unit, Faculty of Tropical Medicine Mahidol University Bangkok Thailand; ^2^ Centre for Tropical Medicine and Global Health, Nuffield Department of Medicine University of Oxford Oxford UK; ^3^ Hospital for Tropical Diseases Oxford University Clinical Research Unit Ho Chi Minh City Vietnam; ^4^ Infectious Diseases Data Observatory, Nuffield Department of Medicine University of Oxford Oxford UK; ^5^ National Center for Parasitology, Entomology and Malaria Control Phnom Penh Cambodia; ^6^ Kinshasa School of Public Health University of Kinshasa Kinshasa Democratic Republic of Congo; ^7^ Indian Council of Medical Research National Institute of Malaria Research New Delhi India; ^8^ University of Health Sciences, Ministry of Health Institute of Research and Education Development (IRED) Vientiane Laos; ^9^ Myanmar Oxford Clinical Research Unit Yangon Myanmar; ^10^ Dev Care Foundation Dhaka Bangladesh; ^11^ Pham Ngoc Thach University of Medicine Ho Chi Minh City Vietnam

**Keywords:** amodiaquine, artemether, drug–drug interaction, lumefantrine, malaria, population pharmacokinetics, triple artemisinin‐based combination therapies

## Abstract

**Aims:**

Resistance to the artemisinins and the artemisinin‐based combination therapy (ACT) partner drugs has developed in Southeast Asia, and artemisinin resistance has also emerged in eastern Africa. Triple ACTs (triple artemisinin‐based combination therapies, TACT), consisting of two partner drugs with different mechanisms of action and similar pharmacokinetic profiles, combined with an artemisinin derivative can help to delay or prevent artemisinin resistance and prolong the useful lifetime of the partner drugs. This study aims to characterize the pharmacokinetic properties of a recommended TACT, artemether‐lumefantrine plus amodiaquine, using data from two large clinical trials.

**Methods:**

We analysed data from two randomized, controlled intervention trials conducted between 2015 and 2020 in one African country and two Southeast Asian countries, in which artemether‐lumefantrine was administered alone (*n* = 443) or together with amodiaquine (*n* = 442) to patients with uncomplicated *P*. *falciparum* malaria. Both studies included a sub‐cohort with dense pharmacokinetic sampling, combined with sparse data in the other patients. Concentration–time data of artemether, dihydroartemisinin, lumefantrine, desbutyllumefantrine, amodiaquine and desethylamodiaquine were analysed using nonlinear mixed‐effects modelling.

**Results:**

Pharmacokinetic models were developed for all drugs and demonstrated good predictive performance and goodness‐of‐fit diagnostics. Coadministered amodiaquine was not a significant covariate on pharmacokinetic properties of artemether‐lumefantrine. Model‐predicted *C*_max_ and AUC (median [95% confidence interval, CI]) for artemether were 256 (159–407) ng/mL and 2850 (1820‐4920) h·ng/mL for artemether‐lumefantrine alone, and 230 (123–391) ng/mL and 2800 (1570‐4570) h·ng/mL for artemether‐lumefantrine‐amodiaquine. For dihydroartemisinin, values were 135 (54.5–214) ng/mL and 1870 (813–3015) h·ng/mL for artemether‐lumefantrine alone, and 116 (40.8–186) ng/mL and 1580 (547–2680) h·ng/mL for artemether‐lumefantrine‐amodiaquine. For lumefantrine, values were 15.2 (2.90–31.3) μg/mL and 600 (275–1230) h·μg/mL for artemether‐lumefantrine alone, and 14.1 (2.72–31.4) μg/mL and 586 (269–1070) h·μg/mL for artemether‐lumefantrine‐amodiaquine. Day 7 concentrations of lumefantrine were 452 (215–1240) and 438 (204–1030) μg/mL for artemether‐lumefantrine alone and artemether‐lumefantrine‐amodiaquine, respectively. All geometric mean ratios (GMRs) for the drug–drug interaction (DDI) effect on key pharmacokinetic parameters of artemether, dihydroartemisinin and lumefantrine fell within the 0.80–1.25 range, with the majority of the corresponding 90% CI also contained within this range. This indicates no clinically relevant DDIs between artemether‐lumefantrine and amodiaquine.

**Conclusions:**

The DDI effect of amodiaquine on the pharmacokinetics of artemether‐lumefantrine is expected to be minimal, the based on the current analysis. However, further large‐scale clinical trials are needed to confirm this finding.

**What is already known about this subject**

- Triple artemisinin‐based combination therapies (TACTs) has emerged as a viable option to delay and prevent artemisinin resistance in malaria and prolong the useful lifetime of the therapy.
- Two recent large clinical trials demonstrated favourable efficacy and safety of artemether‐lumefantrine plus amodiaquine in patients with acute uncomplicated *falciparum* malaria.
- The amodiaquine effect on the pharmacokinetics of artemether‐lumefantrine in two clinical trials was inconsistent, warranting a comprehensive evaluation.

**What this study adds**

- The pooled population pharmacokinetic analysis showed that amodiaquine had no significant effect on the pharmacokinetics of artemether, lumefantrine and their active metabolites.
- The pharmacokinetic exposures of the individual drugs in the TACT arm were compared to that in the ACT arm.

## 1 INTRODUCTION

According to the World Health Organization (WHO) world malaria report of 2024, there were an estimated 263 million malaria cases and 597 000 malaria deaths in 2023.^1^ In the past two decades the treatment of malaria has achieved significant advances with the introduction of artemisinin‐based combination therapies (ACTs) as first‐line treatment for uncomplicated *Plasmodium falciparum* malaria. However, the disease burden caused by malaria is still unacceptably high and new challenges to antimalarial treatment have emerged, particularly the emergence and spread of *P. falciparum* parasites that are resistant to both artemisinins and to the partner drugs used in ACTs. High levels of treatment failure with ACTs have been observed in countries in the Greater Mekong Subregion.^2^ Currently, there are no alternative antimalarial drugs to ACTs that can be deployed across malaria endemic countries as first‐line treatment for uncomplicated *falciparum* malaria. Triple ACTs (TACTs), the combination of a short‐acting artemisinin derivative and two longer‐acting partner drugs, are being developed.^3^, ^4^ Two recently completed clinical trials—Tracking Resistance to Artemisinin Collaboration II (TRACII) and Triple ACTs in Cambodia and Vietnam (TACT‐CV)—assessed the efficacy, safety and tolerability of two TACTs (artemether‐lumefantrine plus amodiaquine and dihydroartemisinin‐piperaquine plus mefloquine). TACTs were found to be efficacious, well‐tolerated and safe treatments of uncomplicated *P. falciparum* malaria, even in areas with artemisinin and ACT partner‐drug resistance.^5^, ^6^ TACTs could extend the useful therapeutic life of existing antimalarial drugs and prevent or delay the emergence of artemisinin resistance.^7^

The theoretical risk of drug–drug interactions (DDIs) between amodiaquine and artemether‐lumefantrine was evaluated based on their metabolic pathways and potential for enzyme induction or inhibition. Artemether is demethylated to dihydroartemisinin by the liver enzymes CYP3A4 and CYP2B6, and is also a CYP2B6 inducer, resulting in time‐dependent pharmacokinetic (PK) properties. Dihydroartemisinin is further glucuronidated by UDP‐glucuronosyltransferase (UGT) 1A9 and 2B7.^8^ It is also a weak CYP1A2 inhibitor. Lumefantrine mainly undergoes CYP3A4 metabolism and inhibits CYP2D6 in vitro.^9^ The pharmacologically active metabolite, desbutyllumefantrine, is conjugated by a uridine‐glucuronosyltransferase (UGT) isoform.^8^ Amodiaquine is mainly metabolized to desethylamodiaquine by CYP2C8, and undergoes minor metabolism through CYP1A1 and CYP1B1.^10^ Further metabolism of desethylamodiaquine to desethylamodiaquine‐quinoneimine has been suggested, mediated by multiple CYP enzymes (CYP2D6, CYP3A4, CYP2C8 and CYP2C9) with similar contribution.^11^ There is no evidence suggesting that either amodiaquine or desethylamodiaquine acts as a strong or moderate inducer or inhibitor of drug‐metabolizing enzymes. Thus DDI are not anticipated because of the independent metabolism pathways of each individual component of the TACT.

The potential DDI between the ACTs and the second partner drugs was investigated in a dense PK cohort in a subset of patients in both TRACII and TACT‐CV, using a non‐compartmental analysis (NCA) approach.^6^ There was some inconsistent evidence for a DDI between artemether–lumefantrine and amodiaquine in the two clinical trials. In the TRACII study, significantly lower peak concentrations of both artemether (*C*_max_ − 24.9%) and its active metabolite dihydroartemisinin (*C*_max_ − 32.0%) were observed in the artemether–lumefantrine plus amodiaquine group, along with a non‐significant decrease in artemether and dihydroartemisinin AUC (−15.9% and −24.6%, respectively). A non‐significant decrease in exposure to both lumefantrine (AUC_T_ − 32·0%) and desbutyllumefantrine (AUC_T_ − 20.0%) after the first dose was also observed, along with significantly lower exposure after the last dose for both lumefantrine (AUC_T,lastdose_ −48·4%) and desbutyllumefantrine (AUC_T,lastdose_ −45.7%), and lower Day 7 plasma lumefantrine concentrations (−17.3%) in the artemether–lumefantrine plus amodiaquine group. In contrast, no significant DDI was observed between lumefantrine and amodiaquine in the TACT‐CV trial, with similar PK exposures (i.e., *C*_max_ and AUC).^5^

The inconsistent DDI results between the two trials warranted further study. The aim of this study was (1) to develop a population PK model for each individual drug of the TACT (i.e., artemether, lumefantrine, amodiaquine and their active metabolites); and (2) to assess the potential DDI effects of amodiaquine on artemether and lumefantrine (from pooled data from both trials).

## 2 METHODS

### 2.1 Study overview

The TRACII trial (NCT 02453308) was a multicentre, open‐label, randomized trial to assess the efficacy, safety and tolerability of two TACTs (dihydroartemisinin–piperaquine plus mefloquine and artemether‐lumefantrine plus amodiaquine) *vs.* standard ACTs. In this trial, a total of 575 patients (children/adults) from seven sites in five countries (Bangladesh, India, Myanmar, Democratic Republic of Congo [DRC] and Lao PDR) were randomized to receive either artemether–lumefantrine alone or artemether‐lumefantrine plus amodiaquine.

The TACT‐CV trial (NCT03355664) was a multicentre, open‐label, randomized trial to assess the efficacy and safety of artemether–lumefantrine plus amodiaquine *vs*. artemether–lumefantrine alone for uncomplicated *P*. *falciparum* malaria in three sites from two countries: western and eastern Cambodia and Vietnam. A total of 310 patients were enrolled and received treatment.

For both clinical trials, written informed consent was obtained from all participants prior to any study procedures. The protocols were approved by the Oxford Tropical Research Ethics Committee and for each site by the relevant institutional review boards, national ethics committee or both.

All patients from both studies were included in the current population PK analysis. The full details of these two clinical trials have been published previously.^5^, ^6^

### 2.2 Dosing regimen

In both trials, eligible patients received artemether–lumefantrine alone or artemether–lumefantrine plus amodiaquine, both administered orally as six doses over 3 days (0, 8, 24, 36, 48, 60 h) and directly observed by the study team. Artemether–lumefantrine was administered either in a fixed dose (bodyweight > 35 kg) or bodyweight‐based dose (bodyweight < 35 kg) according to WHO guidelines^12^ and given with a fatty snack (TRACII) or 80 mL milk (TACT‐CV) to improve the absorption of lumefantrine. The target dose of amodiaquine was 10 mg base per kg/day, given as a split dose twice daily (together with artemether–lumefantrine). The dosing tables can be found in the Tables S1 and S2.

Patients in both trials (except sites in DRC) also received a single gametocytocidal dose of primaquine (0.25 mg/kg) 24 h after the start of study treatment to limit transmission of the parasite.

### 2.3 PK sampling scheme

Dense sampling to describe the PK profiles for lumefantrine, artemether and amodiaquine and their active metabolites desbutyllumefantrine, dihydroartemisinin and desethylamodiaquine, respectively, was performed in a subset of patients in both trials (the first 20 patients in each study drug arm, in both studies). Recruitment of patients for dense PK sample collection was only operationally feasible at one site each in both TRACII (Bangladesh, *n* = 41) and TACT‐CV (Vietnam, *n* = 38) trials. Children with bodyweight < 20 kg were excluded from the dense PK sampling collection.

The dense PK samples were collected at 1, 2, 4, 6, 8, 12, 24, 64 h, and on Days 4, 7, 14 and 28 after the first dose of ACT or TACT, and additionally at 52 h in the TRACII trial. For the remaining patients, PK samples for partner drugs were collected at baseline, Day 7, and at time point of any recurrent infection detected during 42‐day follow‐up. Of note, artemether and dihydroartemisinin plasma concentrations were not quantified for the samples collected on Day 4 and afterwards, due to their relatively short half‐lives.

The PK blood samples were centrifuged at 4 °C within 6 h after sample collection. The plasma for artemether and dihydroartemisinin measurements were obtained and stored at −80 °C within 45 min after collection. The plasma for partner drugs measurement was obtained and stored at −80 °C within 2 h after collection. All samples were shipped to the Department of Clinical Pharmacology at the Mahidol Oxford Tropical Medicine Research Unit, Bangkok, and stored at −80 °C until analysis.

### 2.4 Drug quantification

Plasma concentrations of amodiaquine/desethylamodiaquine, artemether/dihydroartemisinin and lumefantrine/desbutyllumefantrine were measured using validated liquid chromatography–tandem mass spectrometry (LC–MS/MS) assays. The details of the assays for the former two drugs/metabolites have been published previously.^13^, ^14^ The assay for lumefantrine/desbutyllumefantrine was based on a published liquid chromatography‐ultraviolet (LC‐UV) assay,^15^ and modifications described below. In brief, a 100 μL plasma was protein precipitated with acetonitrile‐formic acid 99:1 v/v followed by filtration through a hybrid SPE phospholipid removal 96‐wellplate (Supelco, St. Louis, MO, USA) and the procedure was automated using a Freedom EVO liquid handler system (Tecan, Mannedorf, Zurich, Switzerland). Isotope‐labelled internal standards were used to compensate for any variation in the extraction procedure and matrix effects. The extracted drugs were separated using a Dionex Ultimate 3000 UHPLC (Thermo Fisher, Germering, Bavaria, Germany) equipped with a Zorbax SB‐CN column (Agilent Technologies, Newport, Delaware, USA). An API5000 triple‐quadrupole mass spectrometer and Analyst 1.7 software (ABSciex, Woodlands, Singapore) were used for drug quantification. The lower limit of quantification (LLOQ) was 9.71 ng/mL for lumefantrine and 1.01 ng/mL for desbutyllumefantrine.

All assays were run using the 96‐wellplate format, including a calibration curve and three replicates of quality control samples at low, middle and high concentrations to ensure precision and accuracy for each batch of clinical samples. The total coefficient of variation of all quality control samples were <15% during drug quantification of clinical samples, thus complying with bioanalytical regulatory standards.

### 2.5 Data collection

The following patient information was collected: age, bodyweight, height, sex, enrolment temperature, dose regimen (amount, date, time), biochemistry assay results (albumin, creatinine, total bilirubin, aspartate transaminase [AST], alanine aminotransferase [ALT]), and baseline *P. falciparum* asexual parasite and gametocyte counts at enrolment. In addition, weight‐for‐age Z‐scores (WAZ) for children below 10 years old were calculated based on WHO growth chart reference 2007 using R packages Anthro^16^ and AnthroPlus.^17^ Additionally, treatment outcome, defined as the 42‐day PCR‐corrected adequate clinical and parasitological response (ACPR), was collected.

The PK sampling date and time were collected and recorded. Missing date/time data for dosing and PK sample collection were imputed by protocol date/time. Missing dose amount data were imputed by the amount of the first dose that the patient received. Missing covariate data were imputed by median level for continuous variables, and categorical variables were assigned to most prevalent category in the covariate assessment, if missing data was less than 20%.

### 2.6 Population PK analysis

The PK data of the TRACII and TACT‐CV trials were merged for a population PK analysis. The population PK analysis was performed using the NONMEM software (version 7.5, ICON Development Solutions, Ellicott City, MD, USA), compiled with gFortran (version 4.60). R studio (2023.09.0 Build 463) was used to visualize NONMEM output and model evaluations. The first‐order conditional estimation method including η‐ε interaction (FOCE‐I) was used throughout the model‐building procedure. Perl‐speaks NONMEM (PsN; version 4.6.0), and Pirana (Version 23.1.1) was used for model automation, model record and diagnostics during the model‐building process.

We used two approaches to handle PK concentrations measured below the LLOQ, referred to as the M1 method (discarding all concentrations below LLOQ), and the M3 method (maximizing the likelihood to predict a measured concentration below the LLOQ as censored data).^18^

Here, we used a simultaneous approach to model parent drugs and metabolites for artemether/dihydroartemisinin and amodiaquine/desethylamodiaquine, since the active metabolites were mainly responsible for the anti‐malarial effect. However, lumefantrine is the major contributor to the malaria parasite killing effect compared to its active metabolite, and to avoid the risk of biasing the modelling of the parent drug (e.g., affecting parameter estimates), we therefore used a sequential modelling approach where lumefantrine data were modelled first, then desbutyllumefantrine was modelled with fixed individual parameters for lumefantrine.

All drug molecules were assumed to be eliminated from the central compartment of the PK model. Parent drugs were assumed to be completely metabolized to their metabolites due to identifiability issues with other model structures. One‐, two‐, and three‐compartment disposition models for parent drugs and metabolites were investigated. First‐order absorption and more complex transit absorption models were investigated to describe the absorption phase of parent compounds.

Interindividual variability (IIV) was added exponentially to all parameters, with the assumption of normal distribution with a zero mean and variance ω^2^. Relative bioavailability (*F*) was fixed to unity in the population, allowing for quantification of the IIV in the absorption process. Interoccasion variability (IOV) of each dose event was evaluated on relative bioavailability and absorption paraments (e.g., absorption rate constant and mean transit time [MTT]), with the assumption of normal distribution with a zero mean and variance π^2^. Additionally, site effect is considered to be random and normally distributed, therefore inter‐site variability (ISV) with a zero mean and variance π^2^ was further assessed on the relative bioavailability. Implementation of IOV and ISV were only assessed on the final covariate model to not bias the covariate evaluation. The residual unexplained variability, assumed to be normally distributed with a zero mean and variance σ^2^, was modelled as an additive error on log‐transformed concentrations, which is approximately equivalent to an exponential residual error on an arithmetic scale.

### 2.7 Covariates model

Both statistical significance and biological plausibility were considered in the selection of the covariate model. First, bodyweight was included on all clearance and volume parameters using a conventional allometric function with fixed exponents of 0.75 and 1.0, respectively. Second, the DDI effect of amodiaquine, as a binary categorical covariate, was evaluated on all PK parameters of artemether, dihydroartemisinin, lumefantrine and desbutyllumefantrine. This DDI effect on key primary PK parameters, such as clearance, mean transit time and bioavailability, as well as the secondary PK parameters (e.g., AUC, *C*_max_ and/or Day 7 concentration) were further assessed by a full covariate modelling approach using 500 non‐parametric bootstraps. Lastly, other potential covariates (e.g., malnutrition [WAZ < −2 for children < 10 years and BMI < 18.5 kg/m^2^ for children ≥ 10 years and adults], age and biochemistry assay results) were investigated. The allometric function of bodyweight and DDI effect (if it was significant in the forward inclusion step) were retained and all other covariates were analysed in a stepwise manner with a forward selection (*P* = 0.01, df = 1, ∆OFV = 6.63) and a stricter backward elimination (*P* = 0.001, df = 1, ∆OFV = 10.83). In the final covariate model, covariates that were statistically significant but biologically implausible were not retained in the final model. Biological relevance was assessed based on prior knowledge of the drug's ADME (absorption, distribution, metabolism and excretion) properties and supporting literature.

### 2.8 Model evaluation

Basic goodness‐of‐fit (GOF) diagnostics were used to evaluate potential systematic errors and model misspecification. The predictive performance of the final model was evaluated using a simulation‐based approach (i.e., visual predictive checks [VPC] and prediction‐corrected VPC [pcVPC], *n* = 1000). Additionally, the uncertainty of the final population PK model was assessed using a sampling importance resampling approach (SIR)^19^, ^20^ with results from a non‐parametric bootstrap (*n* = 100) as input. The descriptive summary for PK parameter estimates (median and the 2.5th–97.5th percentile) was calculated and compared with the values obtained in NONMEM.

### 2.9 Assessment of clinical significance of DDI effect

Individual PK exposure parameters, such as *C*_max_, AUC and Day 7 concentration, were derived from post‐hoc empirical Bayes estimates generated using the final population PK model. The clinical significance of DDI effects was assessed using the geometric mean ratio (GMR) and its corresponding 90% confidence interval (CI) of these specific exposure metrics. Clinical significance was defined by fulfilling at least one of the following criteria: (1) a GMR outside the standard bioequivalence range of 0.80–1.25, or (2) evidence of efficacy loss or safety issues attributable to the DDI effect.

### 2.10 Nomenclature of targets and ligands

Key protein targets and ligands in this article are hyperlinked to corresponding entries in http://www.guidetopharmacology.org, and are permanently archived in the Concise Guide to PHARMACOLOGY 2021/22.^21^

## 3 RESULTS

A total of 885 patients with uncomplicated *P. falciparum* malaria, treated with artemether‐lumefantrine (*n* = 443) or artemether‐lumefantrine‐amodiaquine (*n* = 442), were included in this pooled population PK analysis. Of these, 40 patients treated with artemether‐lumefantrine and 39 patients treated with artemether‐lumefantrine‐amodiaquine were subject to dense PK sampling. The baseline characteristics of the overall patients and dense PK cohorts in the two trials, stratified by treatment, are shown in Table 1 and Table S3. The TRACII trial enrolled more young children, resulting in lower median age and bodyweight but higher median dose (mg/kg) and higher baseline *P. falciparum asexual* parasite densities. Other characteristics were generally comparable between treatment arms and studies.

**TABLE 1:** The baseline demographic data of TRACII and TACT‐CV clinical trials.

	TRACII		TACT‐CV	
	AL	AL + AQ	AL	AL + AQ
*N*	289	286	154	156
Male (%)	202 (69.9)	198 (69.2)	142 (92.2)	132 (84.6)
Artemether dose (mg/kg/day)	3.4 (1.6, 5.3)	3.5 (2.0, 5.3)	3.1 (2.0, 5.0)	3.1 (2.1, 5.3)
Lumefantrine dose (mg/kg/day)	20.4 (9.5, 32.0)	20.9 (12.0, 32.0)	18.6 (11.2, 30.0)	18.3 (12.3, 31. 8)
Amodiaquine dose (mg/kg/day)	‐	8.4 (5.2, 12.5)	‐	8.5 (5.1, 12.9)
Age (years)	18.0 (1.9, 65.0)	17.0 (2.4, 62.0)	24.5 (6.2, 58.4)	25.0 (4.0, 55.0)
Bodyweight (kg)	42.0 (9.5, 101.0)	41.5 (9.0, 80.0)	51.8 (16.0, 81.4)	52.2 (11.4, 78.0)
Haemoglobin (g/L)	11.1 (7.0, 14.9)	11.1 (6.5, 15.7)	12.8 (8.1, 17.4)	12.8 (8.3, 17.5)
Haematocrit (%)	36 (22, 51)	37 (19, 55)	40 (27, 54)	39 (28, 50)
Neutrophils (%)	64.0 (16.8, 92.0)	64.0 (15.0, 89.0)	69.7 (23.9, 98.2)	70.1 (22.6, 98.8)
Lymphocytes (%)	29.0 (6.1, 66.6)	29.1 (6.0, 79.8)	17.8 (1.8, 58.9)	17.6 (1.2, 54.6)
Serum creatinine (mg/dL)	0.70 (0.20, 2.70)	0.70 (0.10, 2.10)	0.75 (0.30, 1.46)	0.77 (0.20, 1.71)
ALT (U/L)	22 (1, 127)	22 (1, 125)	25 (8, 269)	25 (3, 202)
AST (U/L)	33 (5, 365)	34 (1, 241)	34 (17, 373)	36 (13, 144)
Total bilirubin (mg/dL)	1.1 (0.2, 4.1)	1.1 (0.2, 4.3)	0.8 (0.1, 3.2)	0.8 (0.1, 4.2)
Alkaline phosphatase (U/L)	248 (13, 1172)	264 (24, 1372)	240 (39, 808)	238 (38, 807)
Pf parasite count (/μL)	47 500 (1, 542 500)	52 500 (1, 557 500)	14 390 (9, 216 300)	21 500 (19, 231 600)
Participants with gametocytes present (%)	15 (5.2)	22 (7.7)	14 (9.1)	16 (10.3)
Baseline temperature ( 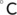 )	37.6 (35.0, 40.5)	37.5 (35.0, 40.2)	37.6 (36.0, 40.9)	37.7 (35.5, 40.7)

*Note*: Continuous variables are presented as median (min‐max).

Abbreviations: AL, artemether‐lumefantrine; AQ, amodiaquine.

### 3.1 Population PK modelling

#### 3.1.1 Artemether

The artemether/dihydroartemisinin model was developed based on all of the available 626 plasma samples (including 42 artemether samples and 101 dihydroartemisinin samples with drug levels measured below the LLOQ), from a total of 79 individuals (41 from TRACII study and 38 from TACT‐CV study) who were enrolled in the dense PK cohorts.

Artemether and dihydroartemisinin plasma concentration–time data were successfully characterized using a joint parent‐metabolite model. Observed concentration–time data were best described by a two‐compartment disposition model for artemether and a one‐compartment disposition model for dihydroartemisinin, with a time‐dependent clearance of artemether (Figure S1). However, this empirical time‐dependent clearance model is not suitable for extrapolation to treatment durations beyond the standard 3 days. The absorption phase of artemether was best described with two transit compartments, with an identical rate constant between compartments. Implementation of bodyweight as a covariate on clearance and volume parameters resulted in a small improvement in model fit (ΔOFV = −0.826), but was retained due to the strong biological basis for this covariate. Coadministration of amodiaquine did not affect the PK properties of artemether or dihydroartemisinin. This was further confirmed by the full covariate approach, which showed that the relative change associated with drug–drug interactions included zero for the main primary PK parameters (Figure S2). Additionally, the secondary PK parameters (i.e., *C*_max_ and AUC) for artemether and dihydroartemisinin, derived from the full covariate approach, largely overlapped between artemether‐lumefantrine alone and artemether‐lumefantrine plus amodiaquine arms (Figure S2). The subsequent covariate search did not find any other significant covariates. Interoccasion variability was added to the mean transit time (ΔOFV = −28.39) and relative bioavailability (ΔOFV = −132.995) resulting in a substantial improvement in model fit. Further assessment of intersite variability on the relative bioavailability resulted in a very small estimate, and was therefore not retained in the final model. The M1 approach adequately handled the data below LLOQ, resulting in no major misspecification of censored data. A further investigation with the M3 approach had a minor impact on parameter estimates and did not improve model diagnostics of censored data, and was therefore not used in the final model.

**FIGURE 1:**
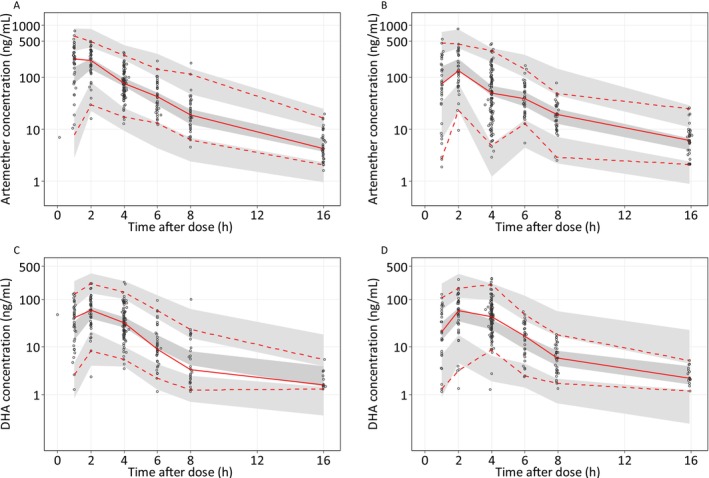
Visual predictive check of the final population PK model for artemether (A, B) and dihydroartemisinin (C, D), stratified by study (A, C for TRACII trial and B, D for TACT‐CV trial). DHA is dihydroartemisinin. The open circles represent the observations, and solid lines represent the 5^th^, 50^th^ and 95^th^ percentiles of the observed data. The shaded areas represent the 95% confidence intervals around the simulated 5^th^, 50^th^ and 95^th^ percentiles.

The VPC, pcVPC and GOF diagnostic plots for artemether and dihydroartemisinin demonstrated a good overall predictive performance of the final model (Figures 1, S3 and S4) and adequate description of the observed data (Figure S4). Furthermore, the SIR results showed that the median parameter estimates were close to the NONMEM estimates, with good precision for all parameters (RSE% < 30%) (Table 2).

**TABLE 2:** Final population pharmacokinetic parameter estimates of artemether and dihydroartemisinin in patients with uncomplicated *P*. falciparum malaria, pooled from the TRACII study (*n* = 41) and TACT‐CV study (*n* = 38).

Parameter	NONMEM	SIR median	CV for IIV/IOV	SIR median	Shrinkage
	Estimates (%RSE)	(95%CI)	(%RSE)	(95% CI)	(%)
**Artemether (ARM)**					
Mean transit time (h)	1.55 (7.3)	1.55 (1.33–1.77)	IOV 54.4 (16.3)	54.2 (46.5–65.7)	
Number of transit compartments	2 Fix				
*F*	1 Fix		IOV 31.6 (22.9)	31.7 (24.7–39.6)	22.3
CL/F _ARM_ (L/h)	79.3 (4.8)	79.2 (71.4–86.6)	21.1 (29.1)	21.5 (16.1–27.7)	14.1
V_C_/F _ARM_ (L)	141 (7.1)	140 (122–161)			
Q/F _ARM_ (L/h)	21.7 (9.32)	21.5 (18.2–26.1)			
V_p_/F _ARM_ (L)	283 (23)	279 (203–446)			
Time dependency on CL	0.551 (16.7)	0.546 (0.387–0.736)			
RUV _ARM_	0.229 (3.8)	0.230 (0.198–0.265)			14.7
**Dihydroartemisinin (DHA)**					
CL/F _DHA_ (L/h)	255 (5.9)	254 (229–288)	41.7 (22.2)	42.2 (33.4–52.0)	58.0
V_c_/F _DHA_ (L)	64.1 (15.3)	64.3 (44.7–82.7)			68.1
RUV _DHA_	0.262 (4.0)	0.261 (0.226–0.306)			14.3

Secondary parameters (median, 95% CI)	AL alone	AL‐AQ	GMR (90% CI)		
*T*_1/2 ARM_ (h)	12.1 (10.0–13.2)	12.2 (11.3–13.5)			
AUC_ARM_ (h·ng/mL)	2850 (1820‐4920)	2800 (1570‐4570)	0.96 (0.84–1.09)		
*C*_max ARM_ (ng/mL)	256 (159–407)	230 (123–391)	0.94 (0.87–1.01)		
AUC _DHA_ (h·ng/mL)	1870 (813–3015)	1580 (547–2680)	0.83 (0.74–0.93)		
*C*_max DHA_ (ng/mL)	135 (54.5–214)	116 (40.8–186)	0.85 (0.77–0.94)		

*Note*: Population estimates are given for a “typical” adult patient weighing 45 kg with acute *P*. *falciparum* malaria. MTT is the mean transit time; CL/F is the elimination clearance; V_C_/F is the central volume of distribution; Q/F is the intercompartment clearance; V_P_/F is the peripheral volume of distribution; *F* is the relative bioavailability; RUV is the residual error variance; AL is artemether‐lumefantrine; AQ is amodiaquine. Time dependent CL is modelled as [(1 + θ*[OCC‐1])*CL], where OCC is the dose occasion from 1 to 6. *T*_1/2_ is the terminal elimination half‐life; AUC is the accumulated area under the concentration–time curve, from time zero to infinity; *C*_max_ is the maximum concentration. Secondary‐parameter estimates are derived from the empirical Bayes post‐hoc estimates. Coefficients of inter‐individual and inter‐occasion variability (IIV and IOV) were calculated as 100 × (e^variance^‐1)^1/2^. Relative standard errors (%RSE) were derived from SIR estimates. *T*_1/2_ of DHA was not reported due to formation‐rate limited elimination. GMR is geometric mean ratio for DDI effect (AL‐AQ *vs*. AL).

**FIGURE 2:**
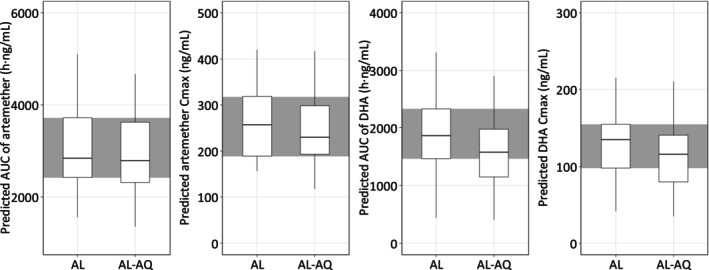
Comparison of PK exposure of artemether and dihydroartemisinin between artemether‐lumefantrine (AL) and artemether‐lumefantrine‐amodiaquine (AL‐AQ) arms. DHA is dihydroartemisinin. The individual PK exposures were derived from the empirical Bayes post‐hoc estimates from the final model. The shaded area represents the 25^th^ to 75^th^ percentiles of PK exposures in the artemether‐lumefantrine alone arm.

The overall PK exposures of artemether and dihydroartemisinin (i.e., *C*_max_ and AUC) were comparable between participants with and without amodiaquine coadministration, as shown in Table 2 and Figure 2. The GMR (90% CI) of DDI effects were 0.96 (0.84–1.09) for AUC, 0.94 (0.87–1.01) for *C*_max_ of artemether, and 0.83 (0.74–0.93) for AUC and 0.85 (0.77–0.94) for *C*_max_ of dihydroartemisinin, respectively.

#### 3.1.2 Amodiaquine

The model for amodiaquine and desethylamodiaquine was based on 352 measurements of parent drug concentrations from a total of 39 patients (21 from TRACII, 18 from TACT‐CV) who were enrolled in the dense PK cohorts, and 725 metabolite concentrations from all available 302 patients (150 from TRACII, 152 from TACT‐CV) who were administered artemether‐lumefantrine‐amodiaquine. Drug levels measured below the LLOQ were excluded from analysis (M1 approach), since there were only a few measurements for amodiaquine (five samples within 96 h post‐first dose) and desethylamodiaquine (15 samples within the follow‐up period of 28 days post‐first dose) below LLOQ.

Some outlier samples showed amodiaquine concentrations measured above the LLOQ at Day 7 after the first dose. Given the short half‐life of amodiaquine (5.2–13.7 h),^22^, ^23^ and to avoid these spurious measurements biasing the modelling results, samples collected at Day 7 and beyond were treated as missing data.

Amodiaquine and desethylamodiaquine plasma concentration–time data were successfully characterized using a joint parent‐metabolite model. Observed concentration–time data were best described by a two‐compartment disposition model for amodiaquine and a three‐compartment disposition model for desethylamodiaquine (Figure S5). The absorption of amodiaquine was adequately described by a first‐order absorption model, with no further improvement in model fit with a transit compartment model. Implementation of bodyweight as a covariate on clearance and volume parameters resulted in a substantial improvement in model fit (ΔOFV = −189.216). The only covariate identified in the covariate search was a difference in amodiaquine or desethylamodiaquine intercompartment clearance between trials (353% higher in the TACT‐CV trial). This covariate was deemed implausible and was not retained in the final model. Interoccasion variability was added on the relative bioavailability (ΔOFV = −36.276) and absorption rate (ΔOFV = −26.552), resulting in a significantly superior model. Further assessment of intersite variability on the relative bioavailability resulted in a very small estimate, and was therefore not retained in the final model.

**FIGURE 3:**
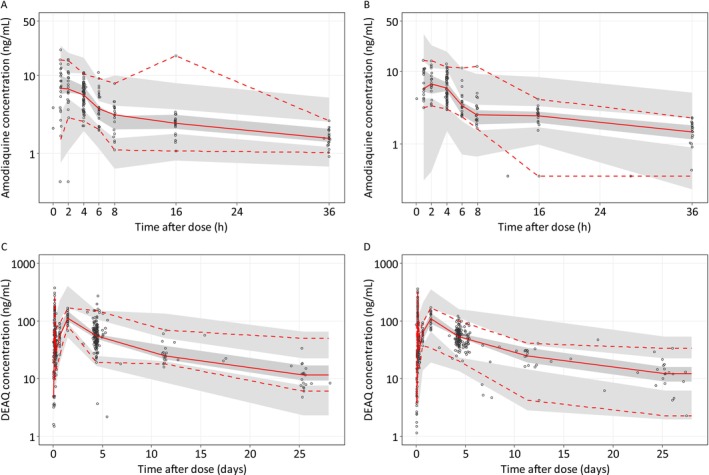
Visual predictive check of the final population PK model for amodiaquine (A, B) and desethylamodiaquine (C, D), stratified by study (A, C for TRACII and B, D for TACT‐CV trials). DEAQ is desethylamodiaquine. The open circles represent the observations, and solid lines represent the 5^th^, 50^th^ and 95^th^ percentiles of the observed data. The shaded areas represent the 95% confidence intervals around the simulated 5^th^, 50^th^ and 95^th^ percentiles.

The VPC, pcVPC and GOF diagnostic plots for amodiaquine and desethylamodiaquine showed good predictive performance in the final model (Figures 3, S6 and S7) and adequate characterization of the observed data (Figure S7). Furthermore, NONMEM parameter estimates were fully contained within the 95% CI from the SIR diagnostics, indicating robustness of the developed model (Table 3).

**TABLE 3:** Final population pharmacokinetic parameter estimates of amodiaquine and desethylamodiaquine in patients with uncomplicated *P*. *falciparum* malaria, pooled from the TRACII study (*n* = 150) and TACT‐CV study (*n* = 152).

Parameter	NONMEM estimates	SIR median	CV for IIV/IOV	SIR median	Shrinkage
	(%RSE)	(95% CI)	(%RSE)	(95% CI)	(%)
**Amodiaquine (AQ)**					
K_a_	1.93 (20.2)	1.92 (1.4–2.91)	IOV: 254 (29.8)	261 (157–560)	68.4
*F*	1 Fix		IOV: 27.5 (15.1)	27.5 (23.2–31.3)	63.2
CL/F _AQ_ (L/h)	2250 (3.2)	2250 (2120‐2390)	11.6 (35.8)	11.8 (7.0–15.4)	74.0
V_C_/F _AQ_ (L)	12 900 (8.6)	13000 (10 700‐15 100)			
Q/F _AQ_ (L/h)	3020 (7.6)	3010 (2640‐3540)			
V_p_/F _AQ_ (L)	27 600 (4.8)	27 700 (25 000‐30 400)			
RUV _AQ_	0.0676 (5.0)	0.0676 (0.0561–0.0827)			15.5
**Desethylamodiaquine (DEAQ)**					
CL/F _DEAQ_ (L/h)	32.2 (3.5)	32.3 (30.1–34.4)	30.6 (21.3)	30.7 (24.0–37.5)	37.8
V_C_/F _DEAQ_ (L)	1260 (8.6)	1260 (1090‐1500)	42.5 (32.2)	42.3 (28.2–57.1)	68.8
Q_1_/F _DEAQ_ (L/h)	117 (17.5)	118 (82.7–161)			
V_p1_/F _DEAQ_ (L)	1640 (15.7)	1630 (1300‐2250)			
Q_2_/F _DEAQ_ (L/h)	37.3 (11.5)	37.5 (29.6–45.7)			
V_p2_/F _DEAQ_ (L)	6440 (7.9)	6420 (5570‐7590)			
RUV _DEAQ_	0.114 (3.7)	0.0996–0.132			15.3
**Secondary parameters (median, 95% CI)**					
*T*_1/2 AQ_ (h)	17.7 (14.3–20.8)				
AUC_AQ_ (h·ng/mL)	1530 (1040‐2050)				
*C*_max AQ_ (ng/mL)	15.1 (10.6–20.5)				
*T*_1/2 DEAQ_ (d)	12.1 (7.7–15.6)				
AUC_DEAQ_ (h·μg/L)	96.5 (53.2–158)				
*C*_max DEAQ_ (ng/mL)	160 (107–232)				

*Note*: Population estimates are given for a “typical” adult patient weighing 45 kg with acute *P*. falciparum malaria. K_a_ is the absorption rate constant; CL/F is the elimination clearance; V_C_/F is the central volume of distribution; Q/F is the intercompartment clearance; V_P_/F, is the peripheral volume of distribution; *F* is the relative bioavailability; RUV is the residual error variance; *T*_1/2_ is the terminal elimination half‐life; AUC is the accumulated total area under the concentration–time curve, from time zero to infinity; *C*_max_ is the maximum concentration. Secondary‐parameter estimates are derived from the empirical Bayesian post‐hoc estimates. *C*_max_ was reported only in the patients with intensive PK collection. Coefficients of variation for interindividual variability and interoccasion variability (IIV and IOV) were calculated as 100 × (e^variance^ − 1)^1/2^. Relative standard errors (%RSE) were derived from SIR estimates.

#### 3.1.3 Lumefantrine

A total of 1448 lumefantrine (96 samples below LLOQ, of those, 93 samples were in absorption phase [<8 h after the first dose]) and 1448 desbutyllumefantrine (319 below LLOQ, of those, 283 samples were in absorption phase [<8 h after the first dose]) plasma concentration measurements from 885 patients (575 from TRACII, 310 from TACT‐CV) were included in the modelling analysis. The M3 approach showed a better predictive performance for absorption phase and censored data compared to the M1 approach, and was therefore used in the model development.

Lumefantrine plasma concentration–time data were best described by a model with two disposition compartments and five transit compartments characterizing the absorption process (Figure S8). Implementation of bodyweight as a covariate on clearance and volume parameters resulted in a substantial improvement in model fit (ΔOFV = −87.52). Coadministration of amodiaquine did not affect the PK properties of lumefantrine. This was further confirmed by the full covariate model, which showed no significant DDI effect of amodiaquine on the main primary PK parameters of lumefantrine (Figure S9). Additionally, the secondary PK parameters (i.e., *C*_max_, Day 7 concentration and AUC) derived from the full covariate approach largely overlapped between artemether‐lumefantrine alone and artemether‐lumefantrine plus amodiaquine arms (Figure S9). The stepwise covariate process identified and retained four significant (*P* < 0.001) covariates, including baseline parasite density, dose (mg/kg) and baseline temperature on relative bioavailability, and a study effect on the central volume of distribution. However, this linear dose–covariate relationship on relative bioavailability is not suitable for extrapolation beyond the dose range studied here. Other potential covariates of interest, such as malnutrition, and biochemical parameters, had no impact on the PK parameters of lumefantrine. Inclusion of interoccasion variability on the relative bioavailability and MTT did not improve model fit, and interoccasion variability was therefore not retained in the final model. Intersite variability was added on the relative bioavailability (ΔOFV = −34.69).

Desbutyllumefantrine plasma concentration–time profiles were best described by a two‐compartment disposition model (Figure S8). Implementation of bodyweight as a covariate on clearance and volume parameters resulted in a substantial improvement in model fit (ΔOFV = −730.44). Coadministration of amodiaquine did not affect the PK properties of desbutyllumefantrine. The full covariate model further confirmed no significant DDI effect on the main primary and secondary PK parameters (Figure S9). Inclusion of age on clearance as a maturation equation improved model fit further (*P* < 0.001), with an estimated age to reach half of full maturation (age 50) at 10.6 years. One additional covariate was identified in the stepwise covariate process; gametocyte density on intercompartment clearance. However, considering its biologically implausible nature, this covariate was not retained in the final model.

**FIGURE 4:**
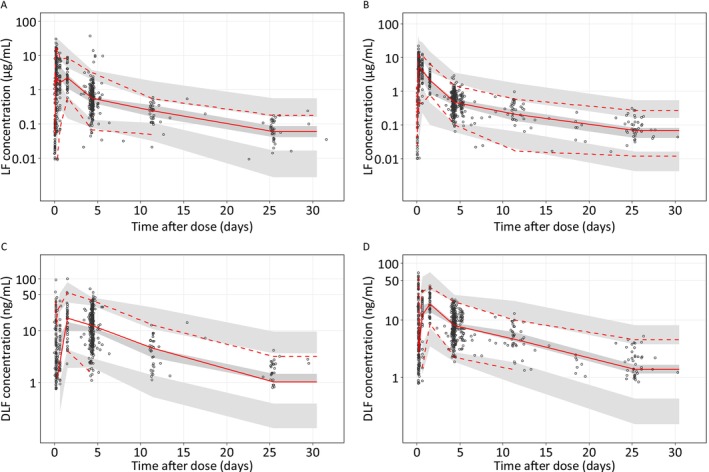
Visual predictive check of the final population PK model for lumefantrine (A, B) and desbutyl‐lumefantrine (C, D), stratified by study (A, C for TRACII and B, D for TACT‐CV trials LF is lumefantrine, DLF is desbutyllumefantrine. The open circles represent the observations, and solid lines represent the 5^th^, 50^th^ and 95^th^ percentiles of the observed data. The shaded areas represent the 95% confidence intervals around the simulated 5^th^, 50^th^ and 95^th^ percentiles.

The VPC, pcVPC and GOF diagnostic plots for lumefantrine and desbutyllumefantrine showed good predictive performance in the final model (Figures 4, S10 and S11). Furthermore, NONMEM parameter estimates were fully contained within the 95% CI from the SIR diagnostics, indicating robustness of the developed model (Table 4).

**TABLE 4:** Final parameter estimates of lumefantrine and desbutyl‐lumefantrine population pharmacokinetics in patients with uncomplicated *P*. *falciparum*, pooled from the TRACII study (*n* = 575) and TACT‐CV study (*n* = 310).

Parameter	NONMEM estimates	SIR median	CV for IIV/ISV	SIR median	Shrinkage
	(%RSE)	(95% CI)	(%RSE)	(95% CI)	(%)
**Lumefantrine (LF)**					
Mean transit time (h)	5.43 (5.8)	5.56 (5.00–6.25)	62.7 (15.7)	58.5 (49.5–72.0)	69.9
Number of transit compartments	5 Fix				
*F*	1 Fix		57.0 (12.3) /13.3 (19.1)	56.9 (49.5–64.9)/ 13.3 (10.3–15.5)	44.6/24.3
CL/F _LF_ (L/h)	4.35 (3.4)	4.35 (4.04–4.61)			
V_C,_/F _LF_ (L)	101 (8.5)	101 (88.0–121)	89.3 (14.3)	89.3 (71.2–103)	64.1
Q/F _LF_ (L/h)	1.65 (6.0)	1.66 (1.45–1.83)			
V_p,_/F _LF_ (L)	311 (5.3)	311 (275–343)			
RUV	0.297 (6.5)	0.304 (0.270–0.344)			24.3
**Desbutyl‐lumefantrine (DLF)**					
CL/F _DLF_ (L/h)	744 (3.5)	721 (677–775)	14.9 (12.5)	15.0 (11.5–18.7)	71.5
V_C_/F _DLF_ (L)	8530 (10.0)	8580 (7170‐10 500)	101 (7.8)	97.5 (77.1–122)	73.6
Q/F _DLF_ (L/h)	1100 (7.5)	1050 (879–1200)			
V_p_/F _DLF_ (L)	62 600 (3.3)	60 900 (56 500‐64 900)			
RUV	0.178 (5.5)	0.177 (0.160–0.199)			0.1
**Covariate relationships**					
Baseline parasite density on *F* (%)	−11.6 (26.4)	−11.6 (−18.0 to −5.6)			
Dose (mg/kg) on *F*	−6.47 (14.3)	−6.54 (−8.50 to −4.82)			
Baseline temperature on *F (%)*	−12.2 (16.4)	−12.4 (−16.2 to −8.43)			
Study effect (TACT) on V_C_/F _LF (%)_	−28.1 (14.7)	−27.7 (−36.3 to −20.0)			
Age on CL/F _DLF_ (year)	10.1 (9.2)	9.8 (8.1–11.7)			

Secondary parameters (median, 95% CI)	AL	AL‐AQ	GMR (90% CI)		
*T*_1/2 LF_ (d)	7.81 (5.65–8.85)	7.75 (5.66–8.70)			
AUC_LF_ (h·μg/mL)	600 (275–1230)	586 (269–1070)	0.95 (0.91–0.99)		
*C*_max LF_ (μg/mL)	15.2 (2.90–31.3)	14.1 (2.72–31.4)	0.82 (0.62–1.07)		
Day 7 LF concentration (ng/mL)	452 (215–1240)	438 (204–1030)	0.94 (0.90–0.99)		
*T*_1/2 DLF_ (d)	5.78 (4.74–9.62)	5.80 (4.79–9.27)			
AUC_DLF_ (h·μg/mL)	4.59 (2.15–10.9)	4.63 (1.79–10.8)	0.97 (0.92–1.01)		
*C*_max DLF_ (ng/mL)	21.4 (4.48–58.6)	22.0 (3.96–46.9)	0.81 (0.62–1.07)		

*Note*: Population estimates are given for a “typical” adult patient weighing 45 kg with baseline parasite density of 10^4.56^ and temperature of 37.5 °C, receiving 9.8 mg/kg dose of lumefantrine. LF, lumefantrine; DLF, desbutyllumefantrine; AL, artemether‐lumefantrine; AQ, amodiaquine. CL/F is the elimination clearance. V_C_/F is the central volume of distribution. Q/F is the inter‐compartment clearance. V_P_/F is the peripheral volume of distribution. F is the relative bioavailability. RUV is the residual error variance. *T*_1/2_ is the terminal elimination half‐life. AUC is the accumulated total area under the concentration‐time curve, from time zero to infinity. *C*_max_ is the maximum concentration. Secondary‐parameter estimates are derived from the empirical Bayesian post‐hoc estimates. Coefficients of variation for interindividual variability and intersite variability (IIV and ISV) were calculated as 100 × (e^variance^ − 1)^1/2^. Relative standard errors (%RSE) were derived from SIR estimates. C_max_ was reported only in the patients with intensive PK collection. Baseline parasitaemia (PBM) was implemented on F as [1 + (θ × (log(PBM)‐4.56)], dose (mg/kg) was added on F as [1 + (θ × (Dose‐9.8)], baseline temperature (TEMP) was included on *F* using [1 + (θ × (TEMP‐37.5)]. Study effect (TACT) was implemented on V_C_/F _LF_ as a proportional equation [1 + θ × TACT]. Age was added on CL/F_DLF_ as [age/(θ + age)]. GMR is geometric mean ratio for DDI effect (AL‐AQ versus AL).

**FIGURE 5:**
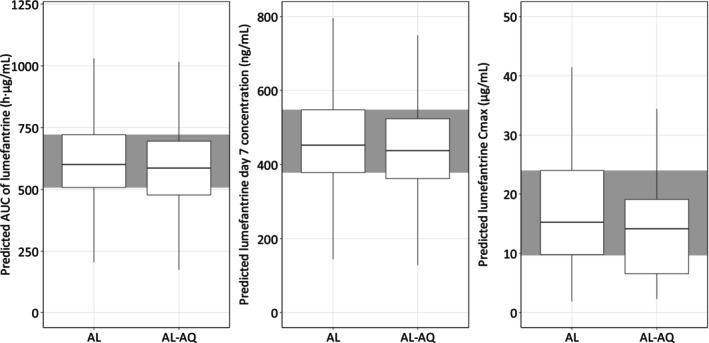
Comparison of PK exposure of lumefantrine between artemether‐lumefantrine (AL) and artemether‐lumefantrine‐amodiaquine (AL‐AQ) arms. The individual PK exposures were derived from the empirical Bayes post‐hoc estimates from the final model. The shaded area represents the 25^th^ to 75^th^ percentiles of PK exposures in the artemether‐lumefantrine arm.

The overall PK exposures to lumefantrine (i.e., *C*_max_, Day 7 concentration and AUC) derived from the final model were comparable between participants with and without amodiaquine coadministration (Table 4 and Figure 5). The GMR (90% CI) of DDI effects were 0.95 (0.91–0.99) for AUC, 0.82 (0.62–1.07) for *C*_max_, and 0.94 (0.90–0.99) for Day 7 concentration.

### 3.2 Comparison of PK exposure between treatment success and failure

Patients who had recrudescent *P. falciparum* infections, i.e. late treatment failures (*n* = 16), had lower PK exposure compared to those with treatment success (*n* = 869). As shown in Figure S12, the mean lumefantrine Day 7 concentration was 447 ng/mL (95% CI, 215–1210) in patients without failure, while the mean Day 7 concentration was much lower in patients with treatment failure (308 ng/mL, 95% CI, 125–553).

### 3.3 Clinical significance of DDI effect

The DDI effect of amodiaquine on the drug exposure to artemether, dihydroartemisinin, lumefantrine and desbutyllumefantrine, described as GMR, fell within the standard bioequivalence range of 0.8–1.25. Additionally, the 90% CI for artemether (AUC and *C*_max_), lumefantrine (AUC and Day 7 concentration), desbutyllumefantrine (AUC) also remained within this range. Only the 90% CI for dihydroartemisinin (AUC and *C*_max_) and lumefantrine (*C*_max_), desbutyl‐lumefantrine (*C*_max_) were slightly outside the 0.8–1.25 range.

Considering the excellent efficacy observed with the TACT in the two clinical studies (98% *vs*. 97% in the TRACII study; 97% *vs*. 95% in the TACT‐CV study), and overall comparable safety profiles indicating the absence of efficacy loss and safety issues attributable to the DDI, the interaction between amodiaquine and artemether‐lumefantrine is not considered clinically significant.

## 4 DISCUSSION

This pooled population PK analysis described the PK profiles of three individual drugs contained in the TACT, artemether‐lumefantrine‐amodiaquine. The DDI effect between artemether‐lumefantrine and amodiaquine was adequately evaluated in the population PK analysis. No significant DDI effects were observed and this supports the dosage regimen used in the clinical trials and provides relevant information to develop a fixed dose combination (FDC) for the TACT.

Potential DDIs are a crucial issue that needs to be addressed during the clinical development of TACTs for malaria therapy. Harmful DDI effects could either increase or decrease PK exposure of individual drugs, in turn, affecting tolerability and safety or treatment efficacy. To address this question, we collected PK samples from all participants, including a subset of patients to collect dense PK data, in two large clinical trials. The pooled population PK analysis based on this larger population is sufficient to characterize any DDI between artemether‐lumefantrine and amodiaquine.

Many factors can cause DDIs in the gastrointestinal absorption phase, including alteration in gastric pH, gastric emptying and intestinal motility, and CYP enzyme and transporter activities. Artemether is a neutral compound with weak chromophores,^24^ suggesting pH‐dependent absorption is unlikely. Lumefantrine is a basic and lipophilic drug with slow intestinal absorption. A study indicated that lumefantrine is a P‐gp substrate, and intestinal absorption could be altered when coadministered with P‐gp inhibitors.^25^ However, amodiaquine has been shown to not exhibit P‐gp inhibitory properties at a concentration range of 10–100 μmol/L.^26^, ^27^ P‐gp inhibition by amodiaquine only occurred at higher concentrations (100–1000 μmol/L), which is far above the concentration associated with clinically applicable doses. DDIs affecting the absorption of lumefantrine due to inhibition of P‐gp by amodiaquine were not anticipated.

Hepatic metabolic enzyme and transporter interactions are the main source of known DDIs. As described above, the metabolic pathways of the three individual drugs show no overlap, with the exception that lumefantrine might decrease the metabolism of desethylamodiaquine by inhibition of CYP2D6. However, this effect, if any, would be small, considering that CYP2D6 is a minor pathway in the metabolism of desethylamodiaquine. Additionally, the role of transporters of the three individual drugs is unclear. A recent physiologically‐based pharmacokinetic analysis had satisfied predictions for these three drugs without considering transporters,^28^ suggesting that any potential effect of transporters (e.g., P‐gp) would be of minimal clinical importance. The potential DDIs due to liver enzyme inhibition and induction between artemether‐lumefantrine and amodiaquine appears to be unlikely.

Non‐compartmental analysis (NCA) of TRACII trial showed a lower PK exposure of lumefantrine in combination with amodiaquine. This finding was most likely a chance result. This difference was not seen when we analysed pooled data from the two clinical trials (*n* = 79 for initial analysis from the TRACII and *n* = 885 for pooled). In addition, we observed slightly different PK behaviour of lumefantrine in patients between the two clinical trials, where the TACT‐CV trial showed higher PK concentrations in the absorption phase than that in the TRACII trial, but similar Day 7 concentrations. The exact cause of this cross‐study difference in the absorption phase was unclear but it could be explained as a study effect in the model, with 30% lower central volume of distribution for the TACT‐CV trial. This study effect might be a result of different manner of administration of the drug, with a fatty snack given in the TRACII trial compared with 80 mL milk given in the TACT‐CV trial, affecting the absorption of lumefantrine with a slight increase in *C*_max_ (12.7 *vs*. 14.7 μg/mL).

The exact timing of the fatty snack/milk might also have an impact on the absorption of lumefantrine. Although patients were advised to take the fatty snack/milk with the dose, the exact time of food intake was not recorded in either trial. A clinical study assessing the effect of food type on lumefantrine bioavailability showed a higher bioavailability in patients receiving pancakes compared to milk (1.11‐fold increase for dispersible tablets and 1.75‐fold increase for crushed tablets).^29^ Another healthy volunteer PK study showed comparable PK exposure when lumefantrine was coadministered with oil‐fortified maize porridge or milk.^30^ Earlier work from our group demonstrated that 90% of maximum absorption was obtained by a minimum of 1.2 g of fat intake.^31^ In the present study, a study effect on central volume had minimal impact on terminal elimination half‐life (182 *vs*. 191 h), Day 7 concentration (444 *vs*. 454 ng/mL) and no change in total drug exposure. As a result, this study effect was not considered to be clinically meaningful. Although the time of food intake was not recorded and different food was used in the two clinical trials, these factors did not appear to significantly affect the PK of lumefantrine. This suggests that the exact food administered and the timing of food intake might be of little importance, as long as enough fat has been ingested to facilitate the absorption of lumefantrine.

In the current analysis, the PK data of each individual drug contained in the TACT were adequately described by the proposed PK models. Here, we introduced a time‐dependent empirical parameter on clearance to describe the time‐varying clearance of artemether due to autoinduction of liver enzyme. In this study, the artemether clearance estimate for a typical patient weighing 45 kg was 79.3 L/h. However, it is not feasible to compare this estimate with the reported clearance values (153 and 429 L/h^32^, ^33^) due to different PK sampling occasions and model hypothesis (e.g., no time‐varying PK component in the literature model). The clearance estimates of dihydroartemisinin for a typical patient was 255 L/h, which was within the range of reported values (200 and 419 L/h).^32^, ^33^ In addition, the PK properties of amodiaquine and its active metabolite desethylamodiaquine are similar to the previously reported values from a pooled population PK analysis,^22^ showing comparable clearance (amodiaquine 2250 *vs*. 2735 L/h; desethylamodiaquine 32.2 *vs*. 30.1 L/h) and half‐lives (amodiaquine 17.7 *vs*. 13.7 h; desethylamodiaquine 291 *vs*. 275 h). Furthermore, the lumefantrine PK properties were largely consistent with a previous pooled PK analysis.^34^ In the current study, we found dose (mg/kg) and baseline parasitaemia both significantly affected the bioavailability of lumefantrine, both of which were also identified as significant covariates in a previous analysis.^34^ Baseline temperature was a significant covariate on bioavailability, with reduced bioavailability in patients with high baseline body temperature. This could be a result of reduced absorption due to malaria illness. In addition, age substantially influenced the clearance of desbutyllumefantrine in an age‐dependent maturation manner, with Age_50_ of 10.6 years old. This finding could be interpreted by the age‐relevant maturation of UGT enzyme (the main metabolism enzyme involved in the metabolism of desbutyl‐lumefantrine) in children (Age_50_ 2.6–10.3 years depending on UGT isoform).^35^ The clearance estimates and derived half‐life of lumefantrine was 4.4 L/h and 187 h for a typical patient weighing 45 kg as defined before, which was comparable to the reported values (5.3 L/h and 150 h).^34^ The clearance of desbutyl‐lumefantrine was higher in the current study (744 *vs*. 298 L/h), and the apparent half‐life was comparable (139 *vs*. 148 h).

The findings of this study are based on a large and diverse study population and likely to be robust. Yet there are potential limitations: (1) the dense PK samples were only collected at one site in each study, increasing the chance of inconsistent results in PK. (2) The timing of the fatty snack was not the same in different trials and not recorded to help explain the observed differences. (3) In both studies, only sparse PK samples were collected after the last dose, together with the short half‐life of artemether, making it difficult to adequately describe autoinduction PK behaviour. (4) Only a few young children under 5 years of age were included in the amodiaquine population PK analysis, so no age‐maturation effect was evaluated on the PK of desethylamodiaquine. (5) Samples were not collected after all doses which makes it challenging to fully characterize the autoinduction of artemether.

## 5 CONCLUSION

In this pooled PK analysis containing all the available randomized evidence on the coadministration of artemether‐lumefantrine with amodiaquine, the DDI effect of amodiaquine on lumefantrine PK is expected to be minimal based on the current analysis. However, further large‐scale clinical trials are needed to confirm this finding.

## AUTHOR CONTRIBUTIONS

R.M.H. and J.D. conducted the analysis and draft the manuscript. J.T. deigned the PK. R.W.v.d.P., M.M., T.J.P., M.H., J.J.C., R.T., L.D., L.v.S., A.D., C.A. and N.J.W. were involved in the design or organization of TACT‐CV, or both, or the training of study staff or data management, or both.

## CONFLICT OF INTEREST STATEMENT

The Mahidol Oxford Tropical Medicine Research Unit (MORU) has received funding for other studies of antimalarial treatment from Shanghai Fosun Pharmaceuticals (Group) Co., Ltd, which manufactures artemisinin combination therapies. We declare no other competing interests.

## Supporting information


**Table S1** Artemether‐lumefantrine dosing schedule.
**Table S2** Amodiaquine dosing schedule.
**Table S3** The baseline demographic data of dense PK cohorts in TRACII and TACT‐CV trials.
**Figure S1** Graphical overview of the structural population PK model of artemether and dihydroartemisinin.
**Figure S2** Covariate effect of coadministration of amodiaquine on the PK of artemether and dihydroartemisinin, using a full covariate model approach.
**Figure S3** Prediction‐corrected visual predictive check of the final population PK model for artemether (A, B) and dihydroartemisinin (C, D).
**Figure S4** Goodness‐of‐fit plots of the final population PK model describing artemether and dihydroartemisinin.
**Figure S5** Graphical overview of the structural population PK model of amodiaquine and desethylamodiaquine.
**Figure S6** Prediction‐corrected visual predictive check of the final population PK model for amodiaquine (A, B) and desethylamodiaquine (C, D).
**Figure S7** Goodness‐of‐fit plots of the final population PK model describing amodiaquine and desethylamodiaquine.
**Figure S8** Graphical overview of the structural population PK model of lumefantrine and desbutyllumefantrine.
**Figure S9** Covariate effect of coadministration of amodiaquine on the PK of lumefantrine, using a full covariate model approach.
**Figure S10** Prediction‐corrected visual predictive check of the final population PK model for lumefantrine (A, B) and desbutyl‐lumefantrine (C, D).
**Figure S11** Goodness‐of‐fit plots of the final population PK model describing lumefantrine (A, B, C) and desbutyl‐lumefantrine (D, E, F).
**Figure S12** Comparison of PK exposure to lumefantrine between treatment success and failure.

## Data Availability

The data supporting the findings of this study are available from the corresponding author upon reasonable request.
