# Supplementary material for: Population pharmacokinetics of artemether–lumefantrine plus amodiaquine in patients with uncomplicated Plasmodium falciparum malaria
Source: Br J Clin Pharmacol. 2025 Oct 6;92(2):589–605. doi: 10.1002/bcp.70301 (PMC12850606; doi:10.1002/bcp.70301)
Supplement: Supplementary file 1 — Table S1 Artemether‐lumefantrine dosing schedule. Table S2 Amodiaquine dosing schedule. Table S3 The baseline demographic data of dense PK cohorts in TRACII and TACT‐CV trials. Figure S1 Graphical overview of the structural population PK model of artemether and dihydroartemisinin. Figure S2 Covariate effect of coadministration of amodiaquine on the PK of artemether and dihydroartemisinin, using a full covariate model approach. Figure S3 Prediction‐corrected visual predictive check of the final population PK model for artemether (A, B) and dihydroartemisinin (C, D). Figure S4 Goodness‐of‐fit plots of the final population PK model describing artemether and dihydroartemisinin. Figure S5 Graphical overview of the structural population PK model of amodiaquine and desethylamodiaquine. Figure S6 Prediction‐corrected visual predictive check of the final population PK model for amodiaquine (A, B) and desethylamodiaquine (C, D). Figure S7 Goodness‐of‐fit plots of the final population PK model describing amodiaquine and desethylamodiaquine. Figure S8 Graphical overview of the structural population PK model of lumefantrine and desbutyllumefantrine. Figure S9 Covariate effect of coadministration of amodiaquine on the PK of lumefantrine, using a full covariate model approach. Figure S10 Prediction‐corrected visual predictive check of the final population PK model for lumefantrine (A, B) and desbutyl‐lumefantrine (C, D). Figure S11 Goodness‐of‐fit plots of the final population PK model describing lumefantrine (A, B, C) and desbutyl‐lumefantrine (D, E, F). Figure S12 Comparison of PK exposure to lumefantrine between treatment success and failure. [file BCP-92-589-s001.docx]

**SUPPLEMENTARY MATERIAL**

**Population pharmacokinetics of artemether–lumefantrine plus amodiaquine in patients with uncomplicated *Plasmodium falciparum* malaria**

*J. Ding, R.M. Hoglund, R.W. van der Pluijm, J.J. Callery, T.J. Peto, R. Tripura, S. Das, N.H. Châu, C. Promnarate, M. Mukaka, L. Dysoley, C. Fanello, M.A. Onyamboko, A.R. Anvikar, M. Mayxay, F. Smithuis, L. von Seidlein, M. Dhorda, C. Amaratunga, M.A. Faiz, H.D.T. Nghia, N.J. White, N.P.J. Day, A.M. Dondorp, J. Tarning*

**Tables and Figures**

Table S1. Artemether-lumefantrine dosing schedule

Table S2. Amodiaquine dosing schedule

Table S3. The baseline demographic data of dense PK cohorts in TRACII and TACT-CV trials

Figure S1. Graphical overview of the structural population PK model of artemether and dihydroartemisinin

Figure S2. Covariate effect of co-administration of amodiaquine on the PK of artemether and dihydroartemisinin, using a full covariate model approach

Figure S3. Prediction-corrected visual predictive check of the final population PK model for artemether (A, B) and dihydroartemisinin (C, D)

Figure S4 Goodness-of-fit plots of the final population PK model describing artemether and dihydroartemisinin

Figure S5. Graphical overview of the structural population PK model of amodiaquine and desethylamodiaquine

Figure S6. Prediction-corrected visual predictive check of the final population PK model for amodiaquine (A, B) and desethylamodiaquine (C, D)

Figure S7. Goodness-of-fit plots of the final population PK model describing amodiaquine and desethylamodiaquine

Figure S8. Graphical overview of the structural population PK model of lumefantrine and desbutyl-lumefantrine

Figure S9. Covariate effect of co-administration of amodiaquine on the PK of lumefantrine, using a full covariate model approach

Figure S10. Prediction-corrected visual predictive check of the final population PK model for lumefantrine (A, B) and desbutyl-lumefantrine (C, D)

Figure S11. Goodness-of-fit plots of the final population PK model describing lumefantrine (A, B, C) and desbutyl-lumefantrine (D, E, F)

Figure S12. Comparison of PK exposure to lumefantrine between treatment success and failure

**Table S1. Artemether-lumefantrine dosing schedule**

| **Artemether-lumefantrine (20/120 mg/tablet)** | | | | | | |
| --- | --- | --- | --- | --- | --- | --- |
| **Weight (kg)** | **Hour 0** | **Hour 8** | **Hour 24** | **Hour 36** | **Hour 48** | **Hour 60** |
| 5 – 14.9 | 1 | 1 | 1 | 1 | 1 | 1 |
| 15 – 24.9 | 2 | 2 | 2 | 2 | 2 | 2 |
| 25 – 34.9 | 3 | 3 | 3 | 3 | 3 | 3 |
| >35 | 4 | 4 | 4 | 4 | 4 | 4 |

**Table S2. Amodiaquine dosing schedule**

| **Amodiaquine (150 mg/tablet)** | | | | | | |
| --- | --- | --- | --- | --- | --- | --- |
| **Weight (kg)** | **Hour 0** | **Hour 8** | **Hour 24** | **Hour 36** | **Hour 48** | **Hour 60** |
| 5 – 14.9 | 0.5 | 0 | 0.5 | 0 | 0.5 | 0 |
| 15 – 24.9 | 0.5 | 0.5 | 0.5 | 0.5 | 0.5 | 0.5 |
| 25 – 34.9 | 1 | 1 | 1 | 1 | 1 | 1 |
| >35 | 1.5 | 1.5 | 1.5 | 1.5 | 1.5 | 1.5 |

**Table S3. The baseline demographic data of dense PK cohorts in TRACII and TACT-CV trials**

|  | **TRACII** | | **TACT-CV** | | |
| --- | --- | --- | --- | --- | --- |
|  | **AL** | **AL + AQ** | **AL** | | **AL + AQ** |
| Number of patients | 20 | 21 | 20 | | 18 |
| Male (%) | 18 (90.0) | 20 (95.2) | 18 (90.0) | | 15 (83.3) |
| Artemether dose (mg/kg/day) | 3.2 (2.6, 4.4) | 3.2 (2.6, 4.0) | 3.1 (2.5, 4.1) | | 3.1 (2.1, 4.3) |
| Lumefantrine dose (mg/kg/day) | 19.2 (15.8, 26.6) | 19.2 (15.4, 24.0) | 18.6 (14.8, 24.6) | | 18.4 (13.0, 26.0) |
| Amodiaquine dose (mg/kg/day) | - | 9.0 (7.3, 11.3) | - | | 8.6 (5.8, 12.2) |
| Age (years) | 26.5 (8.0, 45.0) | 30.0 (16.0, 53.0) | 28.5 (13.8, 58.4) | | 25.4 (12.6, 44.3) |
| Bodyweight (kg) | 50.0 (18.0, 61.0) | 50.0 (40.0, 62.0) | 51.5 (32.5, 65.0) | | 52.3 (37.0, 78.0) |
| Hematocrit (%) | 41 (34, 48) | 43 (33, 51) | 42.50 (33.00, 54.00) | | 40.50 (30.00, 50.00) |
| Neutrophils (%) | 60.5 (45.0, 80.0) | 65.0 (39.0, 87.0) | 67.2 (45.1, 86.5) | | 71.2 (54.4, 82.8) |
| Lymphocytes (%) | 34.5 (13.0, 46.0) | 26.0 (9.00, 52.0) | 18.2(5.0, 26.5) | | 11.9 (7.0, 22.8) |
| Serum creatine (mg/dL) | 0.80 (0.30, 1.10) | 0.80 (0.50, 1.20) | 1.01 (0.75, 1.46) | | 1.04 (0.61, 1.71) |
| ALT (U/L) | 22 (11, 39) | 28 (9, 89) | 28 (16, 58) | | 28 (15, 55) |
| AST (U/L) | 31 (21, 49) | 33 (21, 78) | 26 (17, 62) | | 22 (13, 71) |
| Total Bilirubin (mg/dL) | 1.2 (0.40, 2.80) | 1.30 (0.30, 2.70) | 1.2 (0.8, 3.2) | | 1.33 (0.3, 4.2) |
| Alkaline phosphatase (U/L) | 240 (137, 937) | 263 (182, 616) | 71 (40, 175) | | 61 (38, 123) |
| Pf parasite count (/µL) | 35,000 (4,768-200,000) | 45,000 (5,000-197,500) | 5,597 (469-175,890) | | 6,035 (161-44,135) |
| Participants with gametocytes present (%) | 1 (5.0) | 0 (0) | 4 (20.0) | 5 (27.8) | |
| Baseline temperature ($℃$) | 37.3 (35.0, 39.3) | 38.0 (36.1, 39.5) | 38.2 (36.7, 40.5) | 37.5 (36.9, 39.8) | |

Continuous variables are presented as median (range)

**
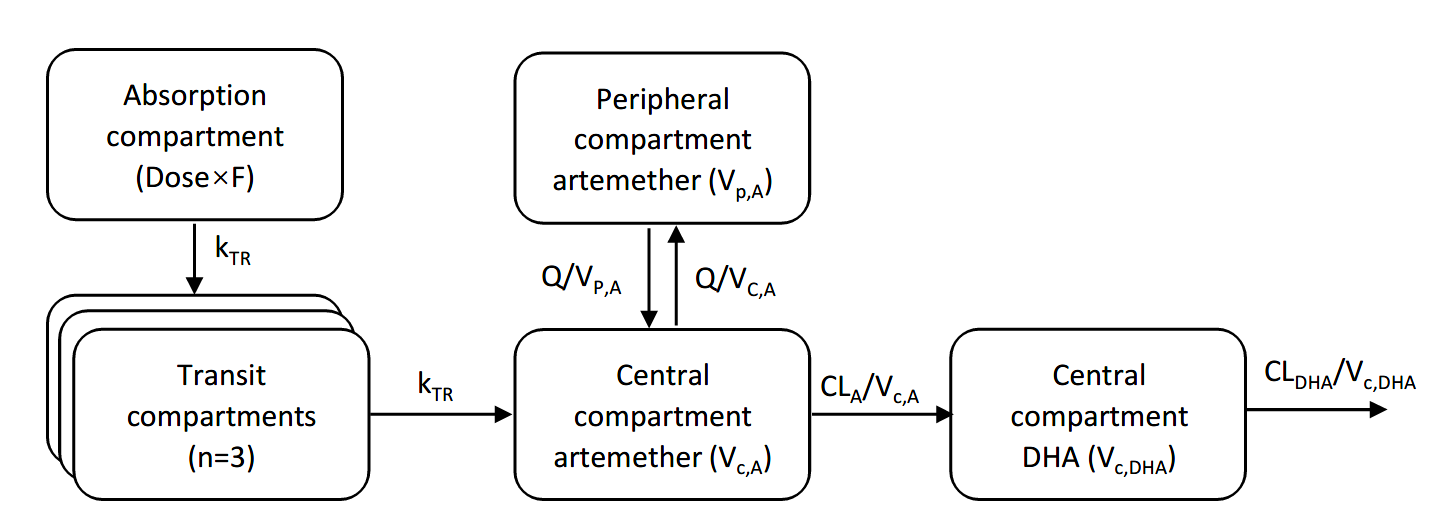
**

**Figure S1. Graphical overview of the structural population PK model of artemether and dihydroartemisinin.**

A, artemether; DHA, dihydroartemisinin; *F*, the relative oral bioavailability; *k*_TR_, transit rate constant; *Q*, inter-compartment clearance; *V*_C_, central volume of distribution; *V*_P_, peripheral volume; CL, elimination clearance.


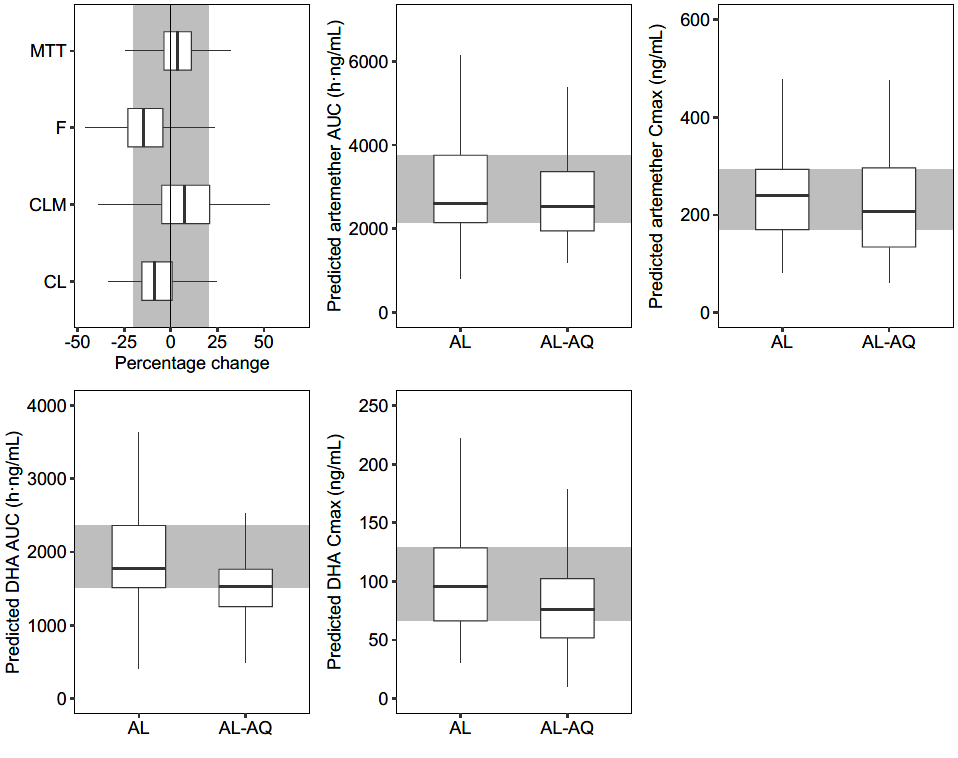
 **Figure S2. Covariate effect of co-administration of amodiaquine on the PK of artemether and dihydroartemisinin, using a full covariate model approach.**

E

D

B

A

C

The results were derived from 500 bootstraps. AL: artemether – lumefantrine. CL, F and MTT are the clearance, relative bioavailability and mean transit absorption time of artemether, respectively. CLM is the clearance of DHA. The shaded area in panel A represents a ±20 % change in parameters. The shaded area in panels B-E represents the 25th to 75th percentiles of PK exposures in the AL arm.

**Figure S3. Prediction-corrected visual predictive check of the final population PK model for artemether (A, B) and dihydroartemisinin (C, D).**

The visual predictive checks were stratified by study; A, C for TRACII and B, D for TACT-CV trial. The Open circles represent the observations, and solid lines represent the 5th, 50th, and 95th percentiles of the observed data. The shaded areas represent the 95% confidence intervals around the simulated 5th, 50th, and 95th percentiles.

**Figure S4. Goodness-of-fit plots of the final population PK model describing artemether and dihydroartemisinin.**

Observed plasma concentrations *vs.* individually predicted concentrations (A, D); conditionally weighted residuals *vs.* population predicted concentrations (B, E); conditionally weighted residuals *vs.* time (C, F). Solid red lines represent locally weighted least squares regressions.

**
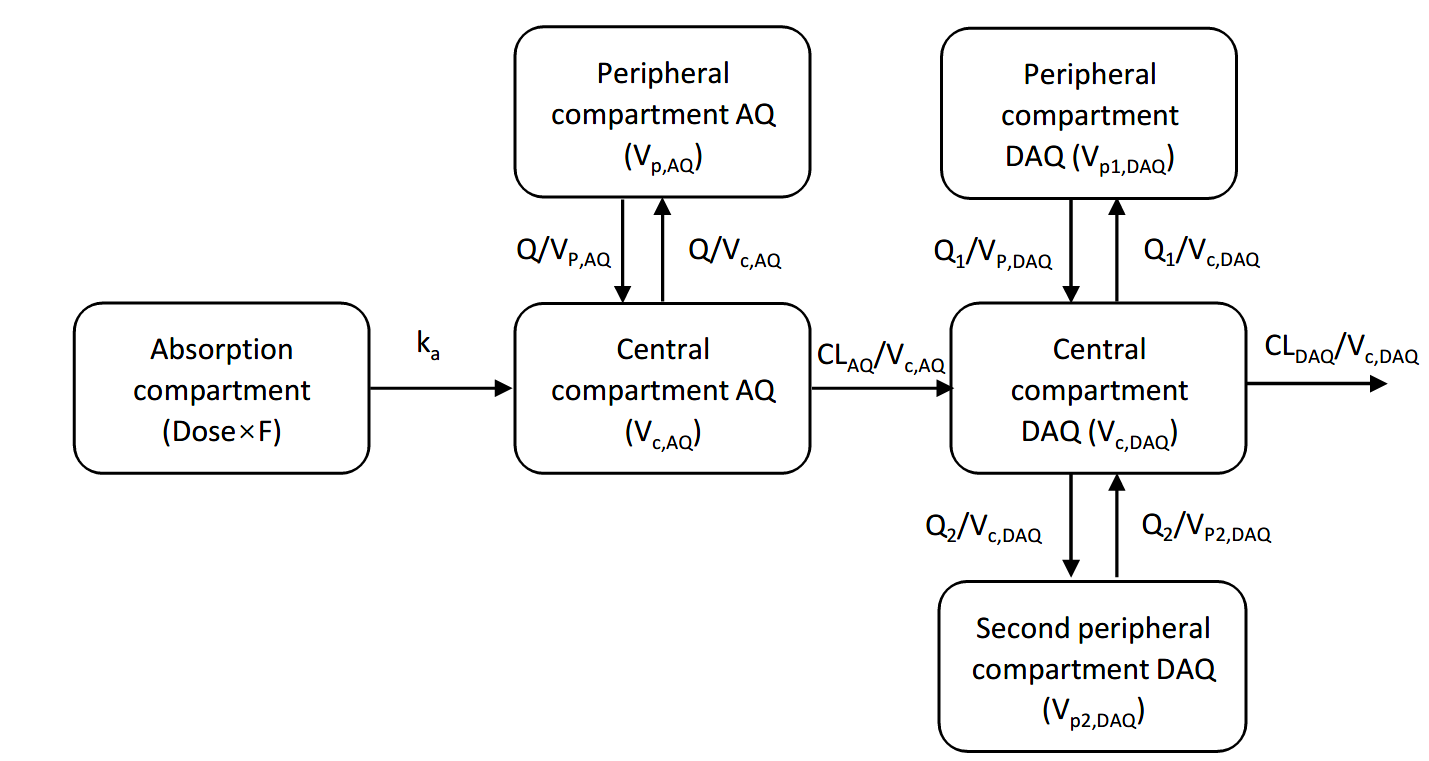
**

**Figure S5. Graphical overview of the structural population PK model of amodiaquine and desethylamodiaquine.**

AQ, amodiaquine; DAQ, desethylamodiaquine; *F*, the relative oral bioavailability; *k*_a_, first-order absorption rate constant; *Q*, inter-compartment clearance; *V*_C_, central volume of distribution; *V*_P_, peripheral volume; CL, elimination clearance.

**Figure S6. Prediction-corrected visual predictive check of the final population PK model for amodiaquine (A, B) and desethylamodiaquine (C, D).**

The visual predictive checks were stratified by study; A, C, E for TRACII and B, D,F for TACT-CV trials. E, F show the first 60 hour. The Open circles represent the observations, and solid lines represent the 5th, 50th, and 95th percentiles of the observed data. The shaded areas represent the 95% confidence intervals around the simulated 5th, 50th, and 95th percentiles.

**Figure S7. Goodness-of-fit plots of the final population PK model describing amodiaquine and desethylamodiaquine.**

Observed plasma concentrations *vs.* individually predicted concentrations (A, D); conditionally weighted residuals *vs.* population predicted concentrations (B, E); conditionally weighted residuals *vs.* time (C, F). Solid red lines represent locally weighted least squares regressions.

**
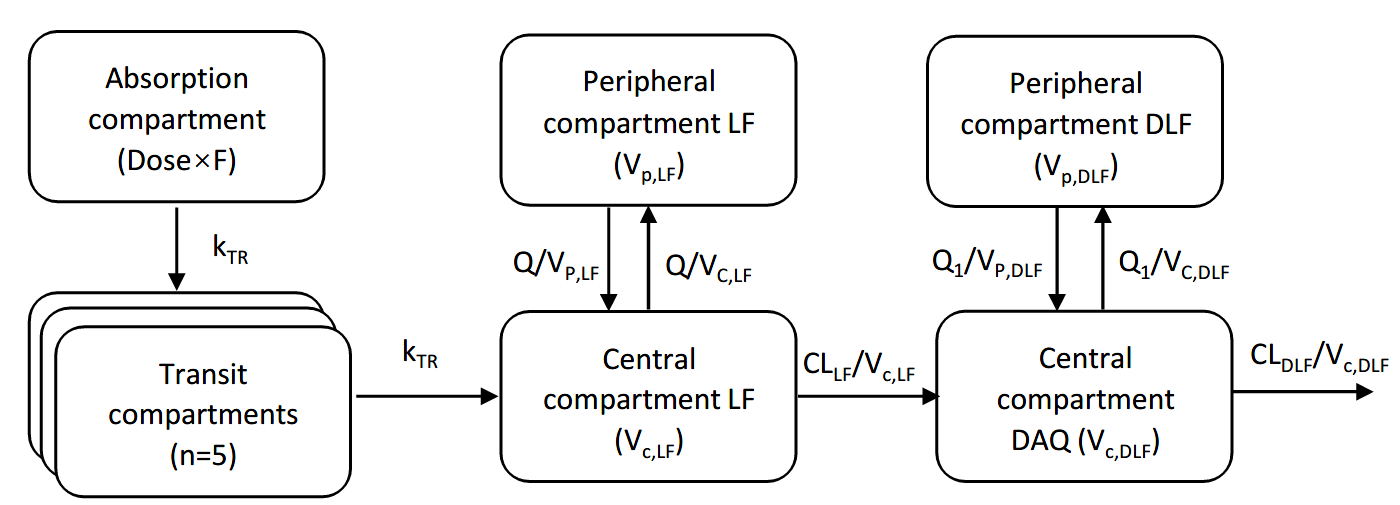
**

**Figure S8. Graphical overview of the structural population PK model of lumefantrine and desbutyl-lumefantrine.**

LF, lumefantrine; DLF, desbutyl-lumefantrine; *F*, the relative oral bioavailability; *k*_TR_, transit rate constant; *Q*, inter-compartment clearance; *V*_C_, central volume of distribution; *V*_P_, peripheral volume; CL, elimination clearance.


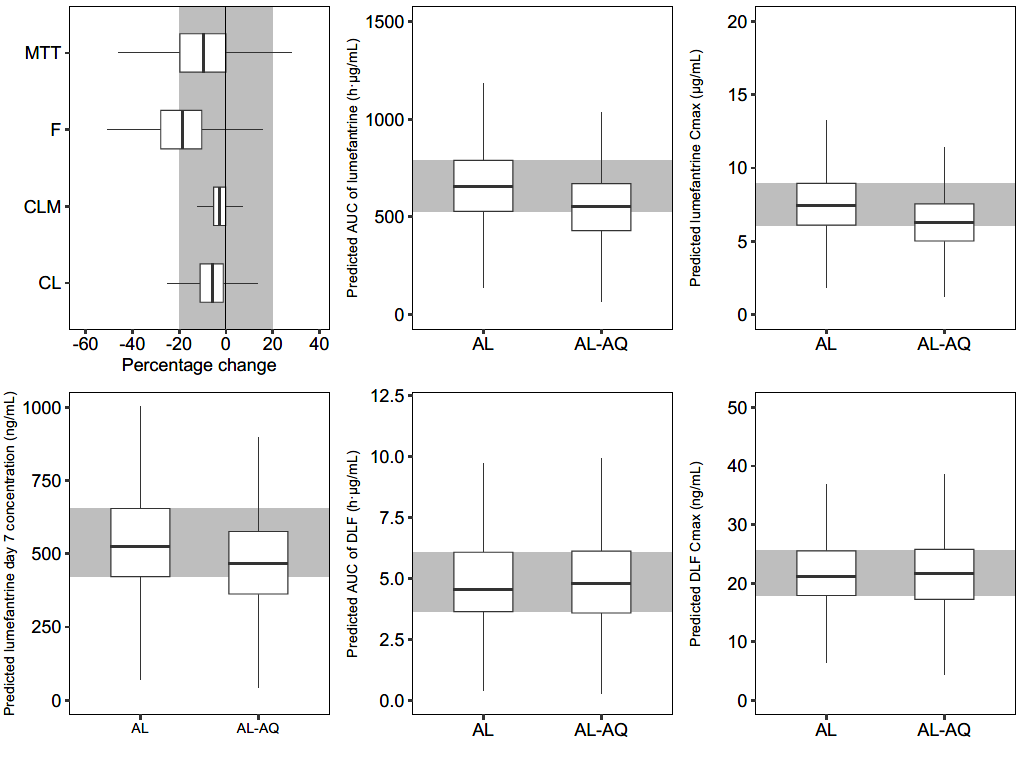


F

E

B

D

A

C

**Figure S9. Covariate effect of co-administration of amodiaquine on the PK of lumefantrine, using a full covariate model approach.**

The results were derived from 500 bootstraps. AL: artemether – lumefantrine, DLF: desbutyl-lumefantrine. CL, F and MTT are the clearance, relative bioavailability and mean transit absorption time of lumefantrine, respectively. CLM is the clearance of desbutyl-lumefantrine. The shaded area in panel A represents a ±20 % change in parameters. The shaded area in panels B-F represents the 25th to 75th percentiles of PK exposures in the AL arm.

**Figure S10. Prediction-corrected visual predictive check of the final population PK model for lumefantrine (A, B) and desbutyl-lumefantrine (C, D).**

The visual predictive checks were stratified by study; A, C for TRACII and B, D for TACT-CV study. The Open circles represent the observations, and solid lines represent the 5th, 50th, and 95th percentiles of the observed data. The shaded areas represent the 95% confidence intervals around the simulated 5th, 50th, and 95th percentiles. The bottom panel show the first 64 hours (for PK concentration) and 12 hours (for fraction censored data).

**Figure S11. Goodness-of-fit plots of the final population PK model describing lumefantrine (A, B, C) and desbutyl-lumefantrine (D, E, F).**

Observed plasma concentrations *vs.* individually predicted concentrations (A, D); conditionally weighted residuals *vs.* population predicted concentrations (B, E); conditionally weighted residuals *vs.* time (C, F). Solid red lines represent locally weighted least squares regressions.

**Figure S12. Comparison of PK exposure to lumefantrine between treatment success and failure.**

The red line, point and bar represented median, mean and standard deviation.
